# Monitoring the Cochlea with Intracochlear Electrocochleography During Cochlear Implantation in Pediatric Patients: Setup, Electrocochleography Patterns and Intraoperative Findings

**DOI:** 10.3390/jcm15145722

**Published:** 2026-07-21

**Authors:** Karolina Haber, Katarzyna Radomska, Adam Blekicki, Józef Mierzwiński

**Affiliations:** 1 Pediatric Cochlear Implant Program, Department of Otolaryngology, Audiology and Phoniatrics, Children’s Hospital of Bydgoszcz, 85-667 Bydgoszcz, Poland; 2 Department of Otolaryngology, Pomeranian Medical University, 71-252 Szczecin, Poland; 3 Department of Otolaryngology, Medicus Clinic, Medicus Sp. z o.o., 50-224 Wrocław, Poland; ablekicki@medicus.com.pl

**Keywords:** electrocochleography, cochlear implant, intraoperative measurements, electrophysiological measurements

## Abstract

**Background/Objectives**: Intracochlear electrocochleography (ECochG) is increasingly used to monitor cochlear function during cochlear implantation, particularly in patients with residual hearing. However, optimal acquisition parameters and interpretation of intraoperative ECochG patterns remain unclear. To (1) establish optimal stimulation and acquisition parameters for intracochlear ECochG, (2) evaluate the feasibility of signal acquisition in pediatric patients, and (3) characterize intraoperative ECochG response patterns in relation to surgical events. Methods: The study consisted of two phases: an optimization phase (*n* = 50) and a clinical observational phase (*n* = 28). In the optimization phase, various stimulation frequencies (500–2000 Hz), recording configurations, and acquisition modes were tested. Based on these findings, a standardized ECochG protocol was developed and applied in the clinical cohort. **Results**: Optimal ECochG recordings were obtained using 500 Hz and 1500 Hz tone bursts at 100 dB nHL, recorded from the apical electrode contact (ICE22) in continuous mode. In the clinical cohort, ECochG responses were detected in 11/28 patients (39%). Cochlear microphonics were observed even in patients without preoperative evidence of residual hearing. Intraoperative signal fluctuations were temporally associated with specific surgical events, including electrode manipulation and suctioning. **Conclusions**: Intracochlear ECochG enables real-time monitoring of cochlear function during implantation. However, interpretation of signal changes remains complex and requires standardized protocols as well as careful consideration of the surgical context.

## 1. Introduction

Electrocochleography obtained through a cochlear implant is increasingly being used to monitor cochlear function during cochlear implant surgery, especially in patients with residual hearing. Electrocochleography (ECochG) is an electrophysiological test that records electrical potentials from the cochlea and auditory nerve in response to sound. ECochG responses can be acquired either extracochlearly or intracochlearly [1,2,3,4,5,6]. ECochG responses can be acquired throughout cochlear implant (CI) insertion either from an extracochlear site or from an intracochlear site via the electrode array contacts as it is inserted [7,8,9,10,11].

For extracochlear ECochG recordings, an electrode is placed on the outer surface of the cochlea, mostly at the round window. These recordings can be obtained before, during, and after electrode insertion. There are currently two approaches to intracochlear recording [11]. In one approach recordings are digitized by the CI and data are reported directly through the device’s telemetry. In the second approach, standard audiologic recording equipment that connects directly to the CI is used [8,11].

ECochG recorded signals have the following components: compound action potential (CAP), summation potential, cochlear microphonics (CM), and auditory neurophonics (ANNs) [2]. The cochlear microphonic (CM) potential is generated primarily by transduction currents in outer hair cells as soon as mechanically gated ion channels open at the tip of stereocilia when the basilar membrane is displaced [12,13]. CM is sensitive to detecting disturbances of cochlear mechanics, such as disruption of the basilar membrane by the electrode array [14].

For stimulation in ECochG, tone bursts or clicks are usually presented via insert earphones [2]. Several animal and clinical studies have confirmed that ECochG recordings in particular provide an opportunity to monitor severe trauma to cochlear structures during implantation [1,2,15,16,17]. As noted by Haumann et al., correlations with postoperative residual hearing still differ strongly, and the relationship remains unclear [2]. However, as suggested by Giardina et al., ECochG is a complex signal that allows us to determine which parts of the signal and which types of changes indicate that trauma is occurring, and whether these changes in response are array-specific [11].

Electrocochleography can provide direct, real-time feedback on electrode insertion and placement within the mastoid cavity. This tool allows surgeons to adjust surgical decisions and specific manipulations based on the observed electrocochleography patterns, potentially reducing surgical trauma.

The purpose of this study was to:

- Specify the setup, stimulation, and acquisition parameters for intracochlear electrocochleography using a research tool provided by Cochlear (Cochlear Ltd., Sydney, NSW, Australia).
- Determine the number of patients in whom an electrocochleography signal was obtained and relate these intraoperative data to preoperative residual hearing.
- Characterize intracochlear electrocochleography patterns/responses depending on events occurring during insertion or electrode placement.

## 2. Materials and Methods

This study consisted of two sequential phases:

An optimization phase (*n* = 50), aimed at establishing the optimal setup and stimulation parameters for intracochlear electrocochleography (ECochG);

A clinical observational phase (*n* = 28), in which the optimized ECochG protocol was applied to evaluate intraoperative responses and their relationship to surgical events and preoperative hearing status.

All procedures were performed in pediatric patients undergoing cochlear implantation. Informed parental consent was obtained for all participants.

All children underwent a standardised preoperative cochlear implant (CI) candidate evaluation. This evaluation included audiometric assessment (subjective and objective measures), vestibular testing, neuroimaging, and medical evaluations by a neurologist, ophthalmologist, and speech and language therapist. A total of 78 children aged 9 months to 3 years meeting the inclusion criteria were enrolled in the study. Fifty were assigned to the parameter-setting group and 28 to the test group.

The clinical observational group consisted of 28 pediatric patients. The mean age was 18 months (range: 9 months to 2 years and 4 months). There were 16 females and 12 males. All patients in both study groups were implanted between January 2024 and January 2026 and received the Cochlear^®^ (Cochlear Ltd., Sydney, Australia) CI632 electrode.

The signal quality in ECochG recordings was assessed based on response reproducibility, signal amplitude, signal-to-noise ratio, and stability during electrode insertion.

A primary goal was to assess intracochlear responses across a broad range of pediatric cochlear implant recipients. Inclusion criteria allowed pediatric CI recipients of any age, deafness etiology, and residual hearing status to participate. Exclusion criteria were inner ear malformations and revision/replacement surgery. The study was conducted in accordance with the Declaration of Helsinki. Ethical review and approval were waived for this study by the Bioethics Committee (Approval No. KB.008.111.2026), as it involved exclusively the analysis of data obtained during standard clinical procedures. All measurements were performed simultaneously with the routine intraoperative assessments that are an integral part of cochlear implantation surgery, without extending the procedure or introducing any additional risk or burden to the patient. Parental informed consent was obtained for all subjects.

Prior to the clinical observational phase, an initial series of 50 cochlear implantations was used to optimize the intracochlear electrocochleography (ECochG) recording setup and stimulation parameters. These procedures were performed in pediatric patients undergoing standard cochlear implantation. The recordings were conducted intraoperatively using the cochlear implant electrode array and the Cochlear Research Platform (version 1.2.1, Cochlear Ltd., Sydney, Australia). The aim of this phase was to determine optimal stimulation frequencies, stimulation intensity, recording electrode configuration, and acquisition mode that provided the most reliable and reproducible ECochG responses. During this phase, ECochG responses were recorded using tone burst stimuli at frequencies of 500, 1000, 1500, and 2000 Hz, presented at an intensity of 100 dB nHL. Continuous recordings were obtained throughout electrode insertion. Different recording configurations were tested, including various intracochlear contacts and reference electrode placements.

Intracochlear ECochG was performed intraoperatively using the CI electrode. The standard electrode array was used in combination with the clinical research software tool. The recording was performed on contact 22 (ICE22), which is the most apical contact. Residual hearing was defined as a preoperative PTA audiogram and ABR or ASSR with at least one threshold better than 90 dB HL at 125, 250, 500, or 1000 Hz.

Anesthesia was induced and a foam plug ear insert connected to a speaker via a sound conductor was placed in the ipsilateral external canal for delivery of acoustic stimulation. Before electrode insertion, a telemetry surgical coil was placed over the skin above the processor, and a laptop established a connection with the implanted device. After preparing the round window or cochleostomy for electrode insertion, a preinsertion measurement of electrode impedances was performed. Recordings were conducted immediately after insertion started, when the apical recording electrode entered the cochlea.

The following rules guided the measurement protocol:

- Before electrode insertion, electrode impedance was measured with the electrode placed in the mastoidectomy cavity filled with saline solution. The aim was to perform this measurement under physical and chemical conditions that most closely resemble the environment (perilymph) present in the cochlea.
- The length of the plastic sound conductor with foam plug placed in the external ear canal was shortened to prevent bending of the tube, which could result in failure to transmit sound from the speaker connected to the sound processor to the patient’s external auditory canal.
- The measurement begins after placing the guide of the Cochlear Nucleus Profile Plus CI632 electrode into the patient’s cochlea, so that recording starts when the ICE22 (apical) electrode is already immersed in perilymph.
- It was established that a reliable electrical response from the cochlea can be considered as a response with an amplitude of at least 5 µV. We assumed that an amplitude below this level may represent a false response, i.e., electrical noise originating from other sources (the patient’s body or electrical devices in the operating room). In some studies, a response amplitude >1 µV was considered sufficient. According to O’Leary et al., of the 61 ears with ECochG responses, amplitudes were <1 μV throughout implantation for 18 ears (23%) and deemed “unclear” for classification [18]. ECochG responses >1 μV in 43 ears (55%) were stable throughout implantation for 8 ears and compromised in 35 ears [18]. The selected threshold was chosen to prioritize recording certainty rather than to define the minimum physiological ECochG response.

Following the development of an optimal electrocochleography protocol, tests were conducted on 28 patients at 500 Hz and 1500 Hz. A detailed analysis was performed on the responses and the stages of electrode implantation: insertion, sealing, and positioning.

The lack of postoperative audiometric outcome data represents a limitation of this study. The primary focus of the present work was the analysis and interpretation of intraoperative ECochG recordings; therefore, postoperative hearing outcomes were not systematically assessed. In addition, in this pediatric cohort, reliable postoperative audiometric evaluation would require repeated testing and, in some cases, additional hospital visits or sedation, which was beyond the scope of the study protocol.

## 3. Results

Optimisation of Recording Parameters.

In the initial cohort of 50 patients, ECochG recordings demonstrated substantial variability depending on stimulation parameters and recording configuration. Among the tested frequencies, responses recorded at 500 Hz and 1500 Hz were consistently characterized by higher amplitudes, better reproducibility, and improved signal stability during electrode insertion compared to recordings obtained at 1000 Hz and 2000 Hz, which frequently showed lower amplitudes and less consistent responses. Continuous recording mode enabled real-time monitoring of changes in cochlear microphonics during electrode insertion and was found to be superior to intermittent measurements in capturing dynamic signal fluctuations. Recordings obtained from the most apical electrode contact (ICE22) provided the most robust and consistent signals, likely due to closer proximity to cochlear regions generating responses to low-frequency stimuli. An intensity of 100 dB nHL was determined to be optimal for eliciting measurable responses in this pediatric population, particularly in patients with limited or absent residual hearing.

Based on these observations, the following parameters were selected for the subsequent clinical phase: stimulation frequencies of 500 Hz and 1500 Hz, stimulation intensity of 100 dB nHL, recording electrode at the apical contact (ICE22), and continuous acquisition mode.

These parameters were chosen as they provided the most consistent and highest-amplitude cochlear microphonic responses across the tested cohort.

Based on these findings, a standardized ECochG protocol was established and subsequently applied in the clinical observational cohort of 28 patients.

A total of 28 measurements were performed after the acquisition parameters had been established. In 17 patients, no ECochG responses were recorded. This group included 2 patients with preoperative residual hearing and 15 patients without residual hearing.

Cochlear microphonics were recorded in 11 out of 28 patients (39%). All of these patients were implanted with the CI632 electrode. Three of them had expected residual hearing. A summary of these findings is shown in Table 1. In 8 patients, cochlear microphonics were recorded despite no residual hearing being expected based on preoperative results.

In 8 out of 11 patients, it was possible to identify specific surgical events that clearly affected the recorded signal [Table S1—Supplementary]. In 3 of them, responses were relatively poor and variable throughout the implantation.

Changes in recorded signal during insertion ECochG patterns recorded in a group of 8 patients are shown in Supplementary Table S1.

## 4. Discussion

Electrocochleography is an emerging tool for monitoring cochlear function during cochlear implant surgery, mostly used as a research tool. However, when electrocochleography is more widely adopted and the interpretation of recorded signals becomes more consistent, it has the potential to become a standard tool providing direct, real-time feedback on electrode insertion, especially in patients with residual hearing.

During cochlear implantation it is convenient to use the CI electrode for intracochlear recordings. The substantial advantage of this method, especially in children, is that no additional invasive measurement electrode is necessary [11]. All measurements can be repeated at any time during and after implantation.

As noted by Giardina et al., another substantial benefit of intracochlear recordings compared to extracochlear recordings is the higher signal-to-noise ratio, which minimizes the number of responses needed to obtain a reliable signal and aids in providing faster feedback to surgeons [11].

ECochG responses and their patterns remain widely discussed. As noted by Sijgers et al., the limited interpretability of ECochG response changes prevents the adoption of this method for clinical use [19].

Several authors have introduced classifications of ECochG response patterns. Harris et al. stratified response tracks into three categories—Type A, B, and C [8]. Type A demonstrated an overall increase in amplitude from the beginning to the end of insertion [8]. Harris’s Type B had a maximal value at the beginning of insertion and a progressive drop throughout insertion, and Type C showed a similar response magnitude at the beginning and end, with a maximal response mid-insertion [8].

Giardina also stratified response tracks into three categories: “overall growth,” “early drop,” and “late drop” [11,12]. In his study, he observed four major patterns of response across all arrays. Campbell et al. presented two response types: “CM preserved” and “CM not preserved” [5].

However, the interpretation of ECochG patterns is more complicated and cannot be generalized to the hypothesis that an increase or stable level throughout insertion indicates no or minimal trauma, whereas a drop in response may indicate trauma [11].

Studies in animals support the possibility that ECochG can be used to detect acute trauma during insertion [11]. Using a rigid electrode designed to penetrate the basilar membrane, intraoperative drops in response magnitude to tones were associated with postoperative histologic trauma [20,21]. However, when a flexible electrode was used, drops in ECochG response magnitude could be reversible when the electrode was withdrawn, and in these cases histological examination verified that basilar membrane integrity was maintained [12,21,22]. The most sensitive metric was a drop in the ongoing response magnitude to a tone, containing the cochlear microphonic (CM) and auditory nerve neurophonic (ANN), rather than changes in the compound action potential (CAP) [11,23].

When discussing ECochG response patterns and cochlear preservation, the initial hypothesis was that increasing or stable responses would indicate an atraumatic insertion, whereas drops in magnitude during insertion would likely indicate immediate insertion trauma. This is the basic metric used in most previous studies. As mentioned above, Campbell et al. reported responses as a binary metric—whether the ongoing response magnitude was preserved or not by the end of insertion, although the criterion for preservation was not stated [5].

Haumann et al. concluded that for intraoperative real-time monitoring, ECochG recordings currently have some drawbacks [2]. They confirmed that a significant drop in intracochlear response patterns may be interpreted as traumatic or atraumatic due to changing relationships with generators as the electrode contact advances [2]. Therefore, one main limitation lies in the change in the recording site during insertion, which was already reported by Calloway et al. [2,10]. Changes during insertion may result both from cochlear trauma and from the changing distance to the signal generator [2]. Nevertheless, sudden drops in amplitude should correspond to cochlear trauma [2].

Giardina et al. also assumed that a response drop mostly indicates mechanical interaction between the electrode and cochlear structures, which might indicate trauma [11]. However, he also demonstrated that response drops can result from interactions between different sources generating ongoing responses [11]. In that case, the response magnitude dropped by nearly 10 dB and then recovered to within 3 dB of baseline as the apical contact moved deeper into the cochlea [11]. It has been reported that hair cell and neural sources responding to the same stimulus frequency can constructively or destructively interfere depending on relative phase and amplitude [11,22,24,25]. In our series, a similar situation occurred in patient 1, when a decrease in response was observed as the apical electrode passed the region most sensitive to 1500 Hz stimulation.

Giardina et al. also observed reversible changes in recorded responses [11]. They suggested that a reversible change could indicate a physical interaction with the membrane, which is not necessarily traumatic [11]. It is possible that the array interacts transiently with the basilar membrane as it slides past the first turn [11].

Moreover, surgical intervention can sometimes recover response amplitude after an initial drop [18,26]. O’Leary et al. concluded in a multi-region, multi-center clinical investigation that CM amplitude drops reflect either interruption of cochlear mechanics or cochlear trauma, but also noted that the transient nature of some drops suggests reversibility in certain cases [18]. It was also possible to recover CM in the study by Bester et al. by withdrawing the electrode array immediately after the drop had occurred [26,27].

In our series, the following events resulted in drops in cochlear microphonics: abrupt removal of the electrode sheath, sealing of the electrode, electrode placement within the mastoid cavity, and suctioning near the cochleostomy due to profuse bleeding. In some cases, after an initial drop in cochlear microphonics, the signals reappeared.

Understanding the intracochlear trajectory of straight, flexible electrodes provides insight into how these electrodes may interact with cochlear mechanics [18,28]. Lateral wall electrodes tend to ride along the lateral cochlear wall, usually at insertion angles beyond 180 degrees, and may contact the basilar membrane [18]. This could explain amplitude drops and their prevalence during the last millimeters of insertion, which coincide with cochlear regions where electrode contacts the basilar membrane [18,28].

This pattern also supports the intervention used in Bester et al. (2021), in which a small withdrawal of the array restored CM amplitude, hypothesized to occur as the array disengages from the basilar membrane [18,26]. Partial withdrawal of the electrode would then reposition the array and release the basilar membrane for normal displacement [18].

Giardina et al. also reported response drops after stylet removal in the CI512 perimodiolar array, a step that allows the array to coil inward toward the basilar membrane, potentially causing mechanical damping or translocation [11].

The main reason for performing ECochG intraoperatively is to provide real-time feedback that may guide surgical interventions aimed at preserving residual hearing [18]. Regarding ECochG patterns and hearing preservation, several studies (Adunka et al., Dalbert, Sim et al., and Radeloff et al.) did not find strong correlations with postoperative residual hearing [2,15,16,27]. Other studies have reported significant correlations with residual hearing (Fitzpatrick et al.) or speech perception (Abbas et al.) [2,29,30]. Giardina et al. showed a strong correlation between ECochG magnitudes and speech perception (46% variability in adults, 36% in older children) [11]. Campbell et al. (2016) found that only permanent changes in CM amplitude (i.e., a CM drop that remained <70% of the previous maximum throughout implantation) were associated with poorer hearing preservation [5,18]. Giardina et al. found that associations with hearing preservation were strongest when phase changes and ANN/CM ratios were considered [11]. Bester et al. provided evidence that using ECochG fluctuations to guide insertion may improve hearing preservation outcomes [26].

ECochG signals cannot be obtained in all patients. According to O’Leary, there are several reasons for this [18]. The first is poor preoperative hearing at the stimulus frequency [18]. The second is poor neurosensory generator function [18]. The third is technical failure related to maintaining acoustic stimulation integrity [18]. He noted that successful electrical stimulation depends not on complete hair cell–nerve connectivity, but on the available neural substrate, i.e., the health of spiral ganglion cells [18]. Giardina et al. suggested that ECochG magnitude reflects cochlear health and resistance to implantation trauma [11]. This is consistent with Wanna, O’Connell et al., who identified lower preoperative thresholds as predictive of better residual hearing outcomes [31].

In our series, we recorded cochlear microphonics in 8 children with no residual hearing detected in ABR/ASSR. In O’Leary et al., in participants with poorer high-frequency hearing, CM remained undetectable until late in the insertion process [18]. In some cases, CM was detectable only after insertion to approximately 20 mm.

ECochG provides different information than the audiogram, as it reflects both hair cell and neural activity. In animal models with high-frequency noise-induced hearing loss, Choudhury et al. demonstrated that basal trauma can affect responses to low-frequency tones generated in more apical regions [20]. These findings indicated that (1) CM is more sensitive than CAP in detecting trauma, (2) responses can be obtained even when functional elements are limited to the apical cochlea, and (3) reductions in response magnitude are a sensitive marker of electrode–tissue interaction [20].

## 5. Conclusions

Preoperatively confirmed and expected residual hearing in cochlear implant candidates is not always reproducible in electrocochleography (ECochG) recordings.

Patients without preoperatively confirmed residual hearing may present cochlear microphonic responses in ECochG; therefore, cochlear implant surgery, especially in pediatric patients, should be performed according to principles of soft surgery, using appropriate electrodes and following a residual hearing preservation protocol.

Surgical maneuvers during electrode insertion may directly influence intracochlear electrocochleography patterns and responses, depending on the events occurring during insertion or final electrode placement.

A standardized protocol based on studies in a larger patient cohort facilitates the interpretation of electrocochleography results and improves the understanding and clinical utility of this valuable intracochlear monitoring technique.

## Figures and Tables

**Table 1:** Characteristics of recorded responses and residual hearing status in a test group.

Group	Number of Patients (N)
Total	28
ECochG responses present	11
ECochG responses absent	17
Residual hearing present	In 2 patients with no recorded response In 3 patients with recorded response
Residual hearing absent	In 15 patients with no recorded response In 8 patients with recorded response

## Data Availability

The data are not publicly available due to privacy restrictions. However, the datasets analyzed during the current study are available from the corresponding authors upon reasonable request.

## Supplementary Materials

The following supporting information can be downloaded at: https://www.mdpi.com/article/10.3390/jcm15145722/s1, Table S1: ECochG patterns recorded in a group of 8 patients.

## Abbreviations

The following abbreviations are used in this manuscript:

ECochG	Electrocochleography
CAP	Compound action potential
CM	Cochlear microphonics
ANN	Auditory neurophonics
CI	Cochlear implant
