# Supplementary material for: Monitoring the Cochlea with Intracochlear Electrocochleography During Cochlear Implantation in Pediatric Patients: Setup, Electrocochleography Patterns and Intraoperative Findings
Source: J Clin Med. 2026 Jul 21;15(14):5722. doi: 10.3390/jcm15145722 (PMC13413122; doi:10.3390/jcm15145722)
Supplement: Supplementary file 1 [file jcm-15-05722-s001.zip › jcm-4357560-supplementary.pdf]

Table S1. ECochG patterns recorded in a group of 8 patients.

No	Residual hearing present/ absent	Intraoperative electrocochleography	Interpretation
1	Absent	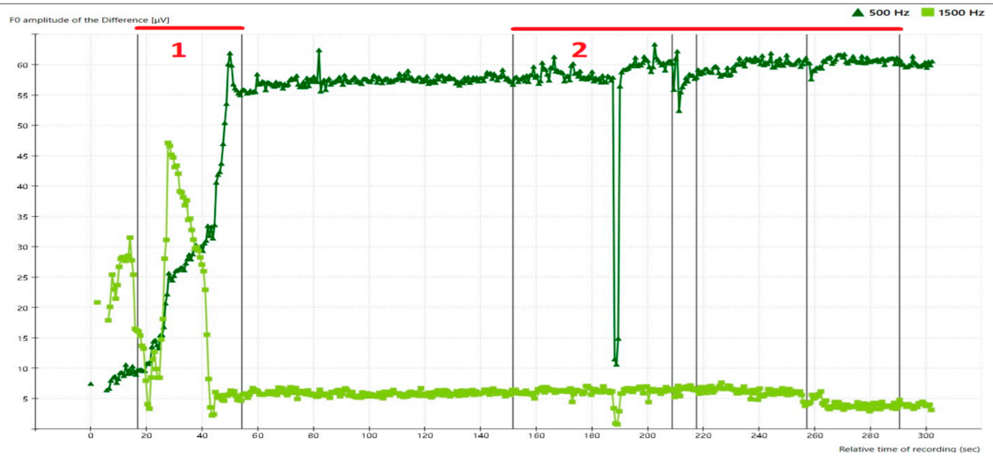	1. Electrode insertion; 2. sealing and securing the intracochlear electrode<br>500 Hz: A clear increase in response was visible from the start of insertion until its completion. There were no noticeable decreases or drops in cochlear microphonics. Sealing and positioning of the electrode had no impact on the signal amplitude.<br>1500 Hz: A clear increase in response was visible from the start of insertion until approximately halfway through, followed by a noticeable decrease. Sealing and positioning of the electrode wires had no additional impact on the response amplitude.<br>**The decrease in response occurred when ICE22 (the apical electrode) passed the region that, according to the tonotopic organization of the cochlea, is most sensitive to 1500 Hz stimulation. This decrease was expected and was not indicative of a traumatic insertion.**

2	Residual hearing at 500Hz	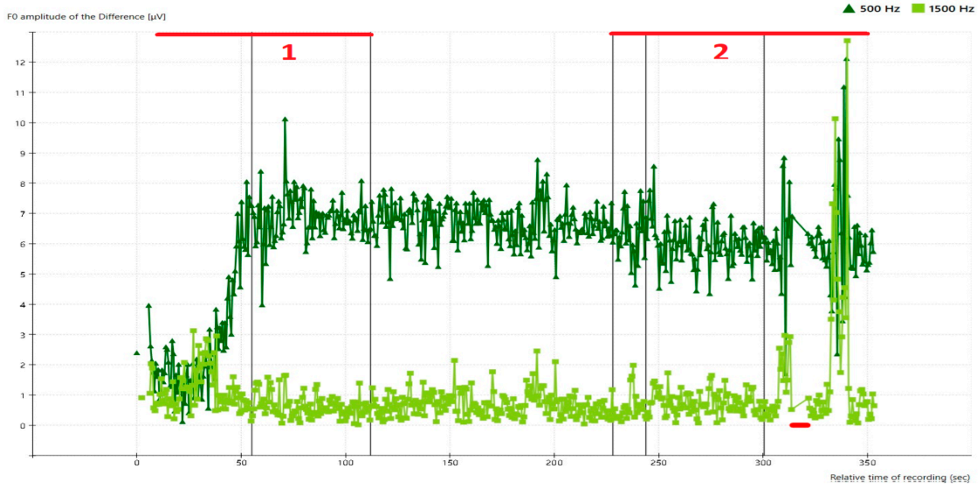	1. Electrode insertion; 2. sealing and securing the intracochlear electrode<br>500 Hz: A clear increase in response was visible from the start of insertion to its completion. There were no significant drops in cochlear microphonics. Sealing and positioning of the electrode had no impact on signal amplitude.<br>1500 Hz: A small increase at the beginning of insertion was followed by a noticeable decrease. The response amplitude reached only about 5 µV at its peak.<br>**The response for 500 Hz also reached only 8 µV at its maximum. It can be assumed that the population of cells sensitive to the presented sound stimulus was poorly preserved in this patient.**
3	Residual hearing at 1000Hz, 2000 and 4000Hz	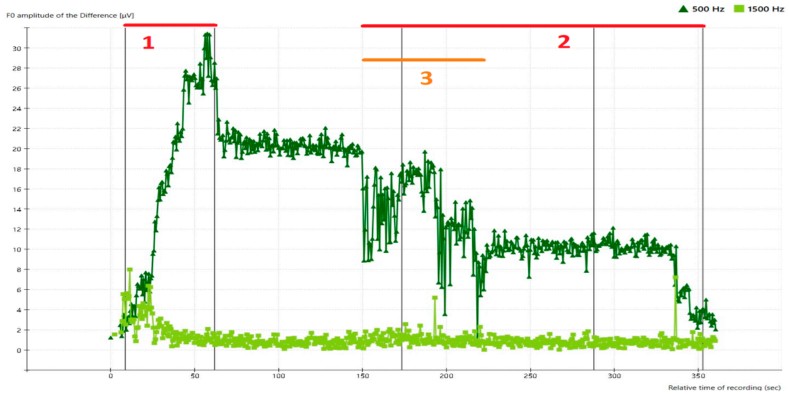	1. Electrode insertion; 2. sealing and securing the intracochlear electrode; 3. suctioning near the cochleostomy due to profuse bleeding<br><br>500 Hz: A clear increase in response was visible from the start of insertion to its completion. After insertion and removal of the electrode sheath, a drop in cochlear microphonics was observed, but the response stabilized at around ~20–22 µV. A sudden drop in cochlear microphonics (3) followed suctioning near the cochleostomy due to profuse bleeding, with stabilization at ~9–12 µV. A further drop in response

			at the end of the measurement may be artifactual and unrelated to the insertion.<br><br>1500 Hz: A small increase at the beginning of insertion was followed by a noticeable decrease. The response amplitude reached about 6 µV at its peak.<br><br>**The initial drop after insertion may be related to overly abrupt removal of the electrode sheath. The difficult surgical conditions – profuse bleeding – and suctioning near the round window had a decisive impact on the amplitude drop at point 3.**
4	Absent	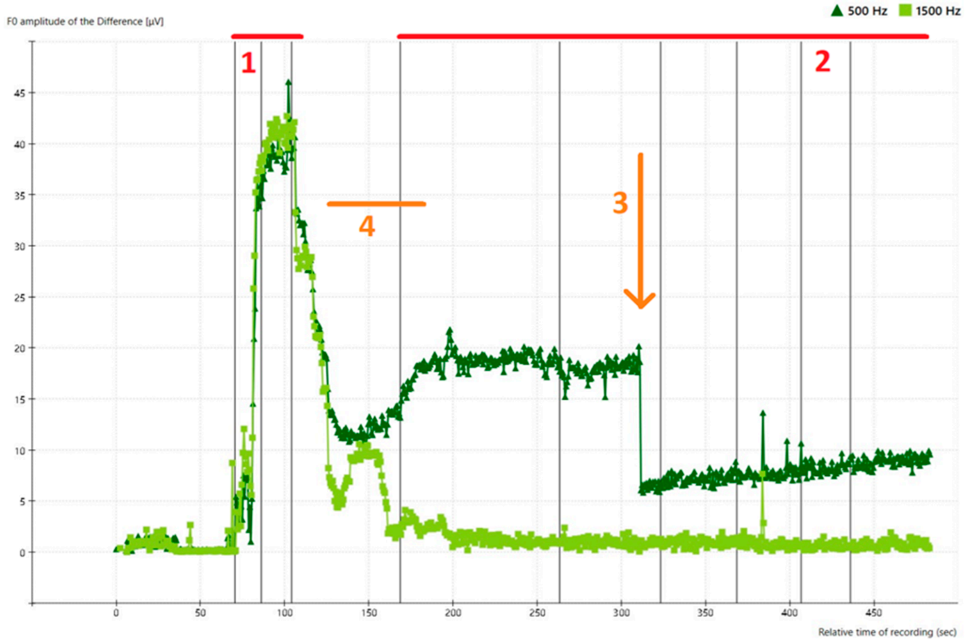	1. Electrode insertion; 2. sealing and securing the intracochlear electrode; 3. sudden drop in CM; 4. after an initial drop in cochlear microphonics, the signals reappeared<br><br>500 Hz: A clear increase in response was visible from the start of insertion to its completion. Sealing and positioning of the electrode had minimal effect on signal amplitude.<br>1500 Hz: A rise was observed at the beginning of insertion, followed by a noticeable decrease, another increase and stabilization below 10 µV. During sealing and electrode positioning, there was a significant drop in response amplitude.<br>It is worth noting that the response amplitude continued to gradually increase until the end of the

			measurement.
5	Absent	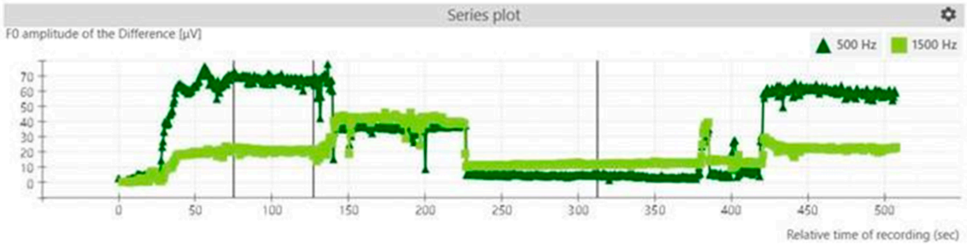	0–130 s: A clear increase in response was visible from the start of insertion and was followed by stabilization of the response for both frequencies.<br>130–225 s: A drop in response was observed for both frequencies.<br>225–420 s: The sound conductor was displaced, which was followed by a sudden drop in the recorded signal for both frequencies.<br>~420 s: Reinsertion of the sound conductor was followed by a stable response similar to those recorded after insertion.
6	Absent	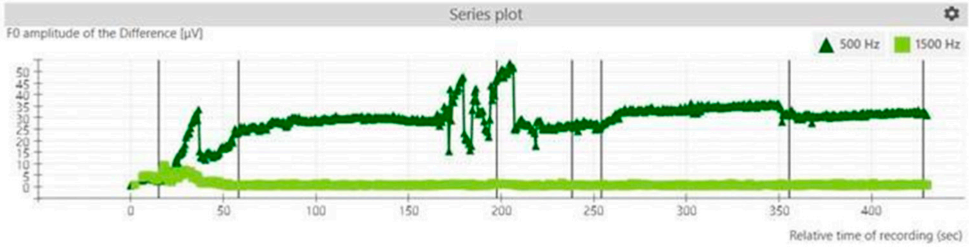	500 Hz: A clear increase in response was visible from the start of insertion. There were no noticeable decreases in cochlear microphonics.<br>1500 Hz: A clear increase in response was visible from the start of insertion, followed by a noticeable decrease noted when the electrode passed the region most sensitive to 1500 Hz stimulation. Between 170 and 210 s after insertion, a sudden drop in cochlear microphonics followed suctioning near the cochleostomy. After the incident, the response stabilized, and no further drops were observed.

7	Residual hearing at 1000Hz	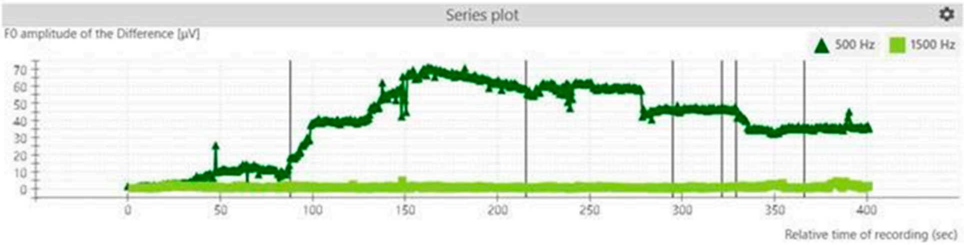	500 Hz: A clear increase in response was visible from the start of insertion. A few drops were noted after 160 s; we assume that the response was affected by sealing and positioning of the electrode. The final response recorded after 330 s was relatively good and stable.<br><br>1500 Hz: A barely noticeable response was recorded.<br><br>It can be assumed that the population of cells sensitive to the presented sound stimulus was poorly preserved in this patient.

8	Absent	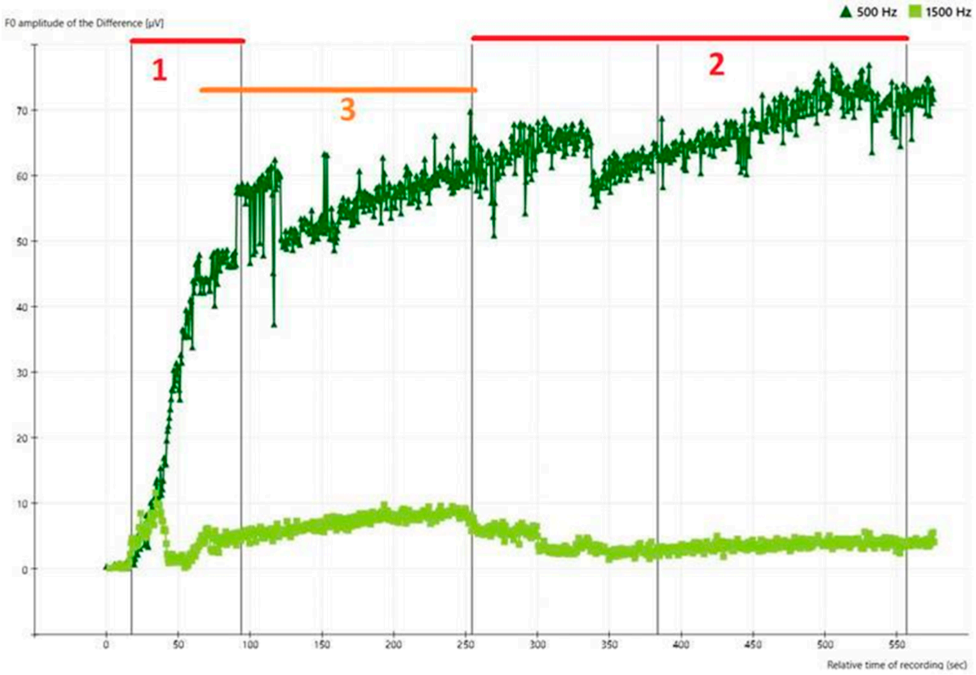	1. Electrode insertion; 2. sealing and securing the intracochlear electrode; 3. sudden drop in CM for 1500Hz<br><br>500 Hz: A clear increase in response was visible from the start of insertion to its completion. There were no noticeable decreases in cochlear microphonics. Sealing and positioning of the electrode had minimal impact on signal amplitude.<br>1500 Hz: A clear increase in response was visible from the start of insertion, followed by a noticeable decrease. Then the signal increased and stabilized below 10 µV. Sealing and positioning of the electrode wires caused an additional drop in response amplitude. At the end of the recording, the response gradually increased.
